# Mosaic Turner Syndrome With Multiple Spontaneous Pregnancies: A Case Report

**DOI:** 10.7759/cureus.53351

**Published:** 2024-01-31

**Authors:** Ayman Altalib, Eman AlSulmi, Danah Bokhari, Zaineb Alhalal, Maram Alismail, Remah Alzayyat

**Affiliations:** 1 Obstetrics and Gynecology, King Fahd Hospital of the University, Khobar, SAU; 2 Medicine and Surgery, Imam Abdulrahman Bin Faisal University, Dammam, SAU

**Keywords:** infertility, pregnancy, mosaic, turner syndrome, case report

## Abstract

Turner syndrome (TS) is an abnormality of the X chromosome affecting females. This genetic defect causes infertility in most cases, but less commonly in patients with the mosaic form of Turner syndrome. In the rare event of a pregnancy, it usually leads to maternal and fetal complications, including miscarriage. In this study, we report a case of mosaic Turner syndrome (45,X/46,XX) in a 34-year-old female who presented to our outpatient clinic with a two-year history of secondary infertility following nine previous spontaneous pregnancies (SP). Her obstetric history showed two successful healthy pregnancies, seven first-trimester miscarriages, one intrauterine fetal demise (IUFD), and one infant death at six months of age. Cases of pregnancy in mosaic Turner syndrome patients are limited and have poor pregnancy outcomes; here, we aim for our case to contribute to the improvement of pregnancy outcomes in such patients.

## Introduction

Turner syndrome (TS) is a common chromosomal disorder caused by the presence of one normal X chromosome and a partial or complete loss of the other X chromosome in affected females [1]. It affects one in every 2,500 female live births [2]. TS can be classified according to the form of X chromosome deletion as follows: 50% of deletions of the X chromosome are of the classic form of TS (45,X), the mosaic form (45,X/46,XX) accounts for 15-25%, and the rest of TS cases have structural abnormalities in the X chromosome [2]. Most TS patients are infertile due to primary amenorrhea and premature ovarian syndrome [3]. However, on rare occasions, some patients with mosaic TS preserve a few follicles and can procreate [1,4]. But usually, these pregnancies are impacted by maternal and fetal complications such as maternal mortality, spontaneous abortions, along with fetal and congenital abnormalities [4]. We present the case of a patient with mosaic Turner syndrome who spontaneously conceived nine times. Only two of the pregnancies were successful, and the rest resulted in miscarriages, intrauterine fetal demise (IUFD), and infant death.

## Case presentation

A 34-year-old female patient, P4+7 L2, presented to the maternal-fetal medicine (MFM) clinic in June 2021 for a postpartum visit after an adverse pregnancy outcome. Her last pregnancy was complicated by intrauterine growth restriction (IUGR), non-immune hydrops fetalis, and fetal cardiac arrhythmia in the form of supraventricular tachycardia (SVT) on a fetal echocardiogram with severely depressed cardiac function and dilated cardiac chambers. Consequently, she began treatment, which led to a normal fetal cardiac rhythm and the resolution of hydrops fetalis. At 33 weeks of gestation, she had a preterm delivery as a result of preterm premature rupture of membranes (PPROM). Unfortunately, the newborn suffered from pneumonia and seizures and later died at six months of age. Concerning other significances in her obstetrical history, she had seven first-trimester miscarriages, one of which required a dilation and curettage (D&C) procedure; four pregnancies continued beyond 20 weeks of gestation; two resulted in healthy children; and the others ended in IUFD.

**Figure 1:**
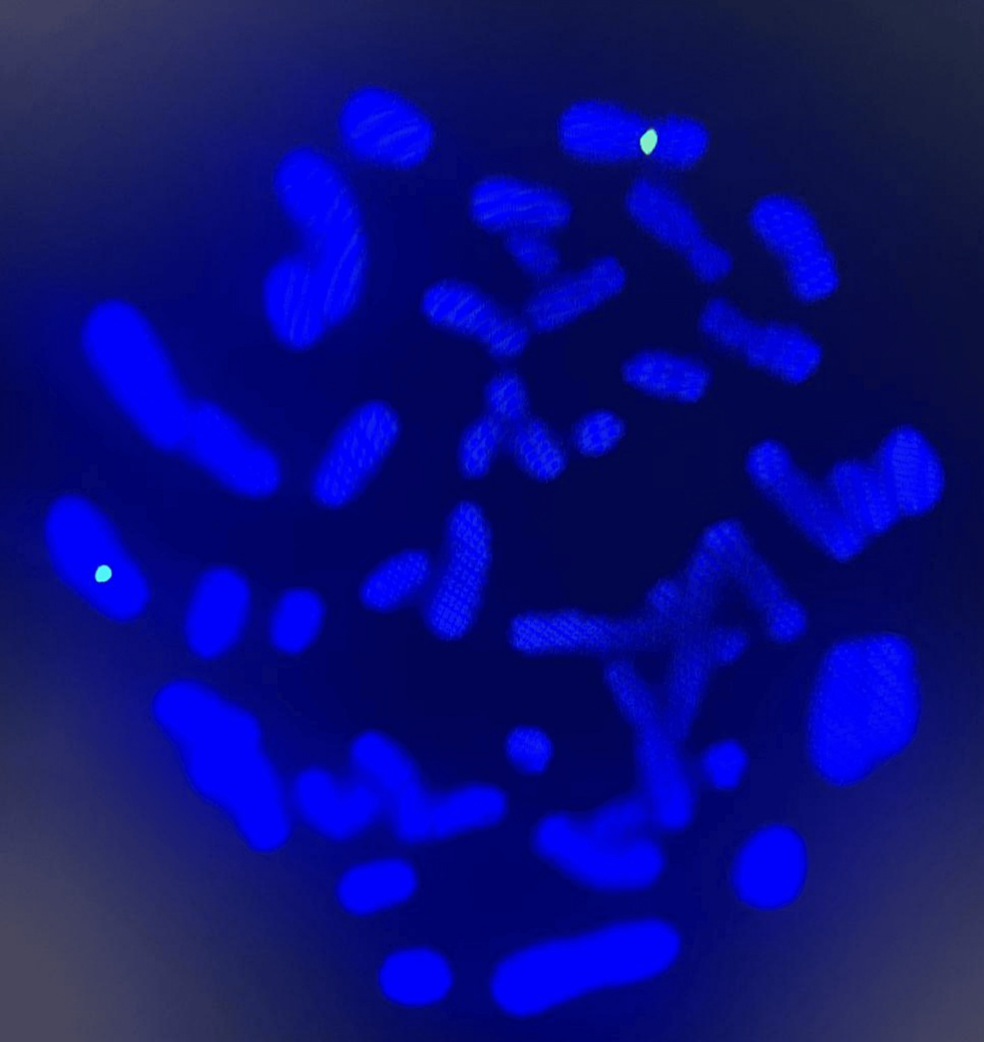
Chromosome analysis Metaphase FISH (fluorescent in situ hybridization) using Vysis SRY/CEP X FISH probe (SpectrumOrange for SRY gene on Yp11.3, SpectrumGreen for DXZ1 alpha satellite sequence specific to chromosome Xp11.1-q11.1) was done on a fixed cell pellet to confirm mosaic Turner syndrome seen in G-banding. The analysis confirms the diagnosis of mosaic Turner syndrome. This image shows a normal female metaphase cell with two copies of chromosome X (two green signals).

**Figure 2:**
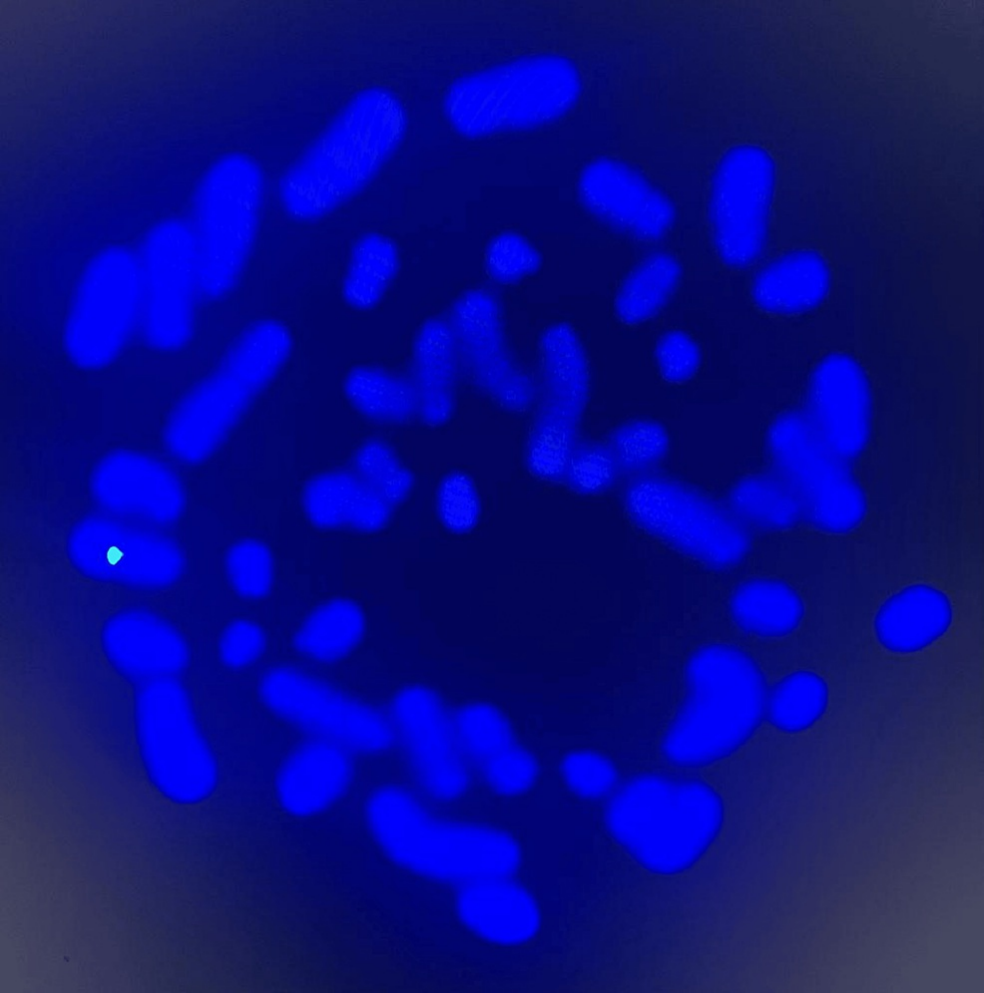
Chromosome analysis Metaphase FISH (fluorescent in situ hybridization) using Vysis SRY/CEP X FISH probe (SpectrumOrange for SRY gene on Yp11.3, SpectrumGreen for DXZ1 alpha satellite sequence specific to chromosome Xp11.1-q11.1) was done on a fixed cell pellet to confirm mosaic Turner syndrome seen in G-banding. The analysis confirms the diagnosis of mosaic Turner syndrome. This image shows an abnormal metaphase cell with one copy of chromosome X (one green signal).

Her medical history included asthma, allergic rhinitis, and bilateral nasal polyps. Physical examination only revealed a short stature of 154 cm. Investigations regarding recurrent early pregnancy losses were normal (including thyroid function tests, lupus anticoagulant, anticardiolipin, protein S, protein C, anti-B2 IgG, anti-B2 IgM, anti-Ro, and anti-La). A chromosomal analysis showed an abnormal fluorescence in situ hybridization (FISH) test result revealing a (45,X3/46,XX17) genotype with three cells of monosomy X out of 20 counted cells (15%) with mosaic TS of low-level mosaicism (Figures 1-2). Her husband is her first-degree cousin, and his karyotype test showed normal 46XY chromosomes. She presented two years later to the MFM clinic with secondary infertility, where she was referred to the Reproductive Endocrinology and Infertility Clinic.

## Discussion

Clinical manifestation

There are typical phenotypes in a TS patient that are usually evident at birth or puberty, which is commonly the time of presentation and diagnosis [5,6]. The clinical features include, but are not limited to, short stature, a webbed neck, widely spaced nipples, and amenorrhea [5,6]. The phenotype of TS is dependent on the different variations of TS in a given patient [6]. This plays a large role in the timing of diagnoses, as some manifestations are not alarming enough to present to a clinic until another manifestation arises later in life, such as infertility [6]. Our case is an example of such late-presenting cases, as the patient’s short stature of 154 cm was not noted until she presented to the infertility clinic, leading to her diagnosis. A retrospective observational study analyzed data from TS females to find an association between genotype, phenotype, and timing of diagnosis [6]. Patients were divided into groups A (classical monosomy 45,X) and group B (all other TS variations). Results showed that most clinical manifestations were earlier and higher in group A, with diagnosis being as early as infancy, while group B had milder and later-appearing phenotypes, which caused a delay in diagnosis [6].

Fertility

Fertility and pregnancy are topics of significance in TS, as it is known that monosomy patients of 45,X have gonadal dysgenesis due to accelerated follicular atresia [5]. Previous literature reviews on spontaneous pregnancies (SP) in TS have presented multiple case reports of 45,X TS patients who were able to birth healthy newborns [7,8]. However, the percentage of monosomy TS patients with SPs compared to other chromosomal variations of TS was notably lower [7,9]. In mosaic TS patients, follicular atresia is slowed, with an increased percentage of chromosomally normal cell lines [5]. Despite this, an interesting finding in one of the literature reviews was that despite the increased possibility of SP for mosaic TS patients, the chances of congenital anomaly are higher than those of 45,X monosomy TS patients [9].

Reproductive options

Considering the difficulty of sharing information with the patients themselves and their parents about the high possibility of future infertility, it is not surprising that parents may often feel that they do not have adequate knowledge to discuss fertility issues with their daughters [10]. This situation is exacerbated by the social stigma of infertility, the family’s wish for their daughter to conceive, and their desire to have a biological grandchild [10]. Due to these challenges and to maximize the benefits of fertility preservation (FP), it is important for medical professionals to evaluate TS patients and facilitate fertility-related discussions and reproductive options as soon as possible in childhood, as the majority of TS women will have a depleted ovarian reserve before puberty [11]. A comprehensive review study has reviewed various FP techniques for women with TS, including vitrification of mature oocytes, oocyte cryopreservation, ovarian tissue cryopreservation (OTC), and embryo cryopreservation [11]. Although vitrification of mature oocytes is the preferred technique of FP, OTC is commonly used in young girls before they reach puberty [11]. In vitro fertilization (IVF) is the assisted reproduction technique that is most used in women with TS [11]. However, our patient had conceived spontaneously.

Pregnancy-related complications

TS patients are more susceptible to comorbidities during pregnancy. Abortion is reportedly experienced in a range between 30.8% and 67.3% of SPs in the first trimester [7,12,13], and, in one review, was mostly found in a mosaic 45,X/46,XX patients [9]. Preterm births above 32 weeks were evidently increased in TS patients in one cross-sectional study [14]. Numerous case reports have shown that pregnancy-induced hypertensive disorders such as pre-eclampsia were found to be more prevalent in TS pregnancies, and they are increasingly reported in oocyte donation (OD) pregnancies [13,15]. Interestingly, the retrospective cohort of Calanchini et al. found that pre-eclampsia was diagnosed in 11% of 156 pregnancies, compared to none in OD pregnancies [12]. The increased prevalence of hypertensive disorders in pregnancies goes hand-in-hand with the results of a cross-sectional study where the risk of ischemic placental disease was 3.2 times higher in TS patients compared to non-TS [14]. Cesarean section (CS) is reportedly increased in TS pregnancies, reaching 46.7% of patients in a cohort study [13]. There are reports revealing that acute preterm CSs were due to maternal cardiovascular complications such as aortic dilatation [7].

Neonatal outcomes

The outcome of TS pregnancies mostly ends in healthy live births [14]. A cross-sectional study calculated a 3.6-fold increased risk of neonatal morbidity in TS patients [14]. Mavridi et al. reviewed four large studies and calculated a live-born rate of around 69.82%. Out of the four studies reviewed, only one included three terminations of pregnancy due to trisomy 21 and early rupture of membranes at 20 weeks [7,13]. Congenital malformations such as cerebral palsy, cleft lip/palate, hydrocephalus, ambiguous genitalia, and TS were present in 11 infants across 160 TS patients’ pregnancies, two of whom were TS patients in the literature review by Mavridi et al. [7]. In a review of 185 TS pregnancies, eight resulted in stillborn births, six infants had congenital anomalies, three had Down syndrome, and 27 had 45,X TS [9]. Infants that are small for their gestational age were reported in 31.8% of patients [14]. This emphasizes the significance of perinatal care and education for TS patients regarding possible pregnancy options and outcomes.

## Conclusions

Turner syndrome is a chromosomal disorder that clinically presents with characteristics such as short stature, a webbed neck, and infertility. The mosaic form of TS may bypass many of the known TS traits, causing a delay in diagnosis similar to that of our patient. Although pregnancy may be achieved in mosaic TS patients, it can be accompanied by complications that can hinder the completion of a healthy pregnancy. Our patient was able to have two healthy, living children among 11 pregnancies. By sharing our case, we hope to raise awareness of Turner syndrome and its connection to pregnancy complications. We would also like to draw attention to the possibility of the mosaic form of TS as a differential diagnosis in patients experiencing fertility issues but not showing characteristic clinical manifestations of TS.
